# SBRT for the Primary Treatment of Localized Prostate Cancer: The Effect of Gleason Score, Dose and Heterogeneity of Intermediate Risk on Outcome Utilizing 2.2014 NCCN Risk Stratification Guidelines

**DOI:** 10.3389/fonc.2014.00312

**Published:** 2014-11-11

**Authors:** Matthew Bernetich, Caspian Oliai, Rachelle Lanciano, Alexandra Hanlon, John Lamond, Stephen Arrigo, Jun Yang, Michael Good, Jing Feng, Royce Brown, Bruce Garber, Michael Mooreville, Luther W. Brady

**Affiliations:** ^1^Rowan University School of Osteopathic Medicine, Stratford, NJ, USA; ^2^Philadelphia CyberKnife Center, Delaware County Memorial Hospital, Havertown, PA, USA; ^3^Department of Radiation Oncology, Drexel University College of Medicine, Philadelphia, PA, USA; ^4^Office of Nursing Research, School of Nursing, University of Pennsylvania, Philadelphia, PA, USA

**Keywords:** stereotactic body radiation therapy, prostate cancer, CyberKnife, hypofractionation, NCCN guidelines, risk stratification

## Abstract

**Purpose:** To report an update of our previous experience using stereotactic body radiation therapy (SBRT) for the primary treatment of prostate cancer, risk stratified by the updated National Comprehensive Cancer Network (NCCN) version 2.2014, reporting efficacy and toxicity in a community hospital setting.

**Methods:** From 2007 to 2012, 142 localized prostate cancer patients were treated with SBRT using CyberKnife. NCCN guidelines Version 2.2014 risk groups analyzed included very low (20%), low (23%), intermediate (35%), and high (22%) risk. To further explore group heterogeneity and to comply with new guidelines, we separated our prior intermediate risk group into favorable intermediate and unfavorable intermediate groups depending on how many intermediate risk factors were present (one vs. > one). The unfavorable intermediate group was further analyzed in combination with the high risk group as per NCCN guidelines Version 2.2014. Various dose levels were used over the years of treatment, and have been categorized into low dose (35 Gy, *n* = 5 or 36.25 Gy, *n* = 107) and high dose (37.5 Gy, *n* = 30). All treatments were delivered in five fractions. Toxicity was assessed using radiation therapy oncology group criteria.

**Results:** Five-year actuarial freedom from biochemical failure (FFBF) was 100, 91.7, 95.2, 90.0, and 86.7% for very low, low, intermediate and high risk patients, respectively. A significant difference in 5 year FFBF was noted for patients with Gleason score (GS) ≥8 vs. 7 vs. 5/6 (*p* = 0.03) and low vs. high dose (*p* = 0.05). T-stage, pretreatment PSA, age, risk stratification group, and use of ADT did not affect 5-year FFBF. Multivariate analysis revealed GS and dose to be the most predictive factors for 5-year FFBF.

**Conclusion:** Our experience with SBRT for the primary treatment of localized prostate cancer demonstrates favorable efficacy and toxicity comparable to the results reported for IMRT in literature. GS remains the single most important pretreatment predictor of outcome.

## Introduction

The American Society of Radiation Oncology (ASTRO) model policy update of 2013 (1) acknowledged that stereotactic body radiation therapy (SBRT) is equivalent to standard radiation modalities used to treat prostate cancer such as intensity modulated radiation therapy (IMRT). In addition, the National Comprehensive Cancer Network (NCCN) (2) “cautiously considers SBRT as an alternative radiation treatment strategy to conventional fractionated regimens at clinics with appropriate technology, physics and clinical expertise” (p.707). For low and intermediate risk prostate cancer, recent published literature (3–6) supports these recommendations with excellent 5 year biochemical control rates and correspondingly acceptable rates of toxicity.

Prognostic factors necessary to guide staging and treatment options for prostate cancer include Gleason score (GS), initial PSA, and T-stage. These factors have been used to group patients into risk categories of low, intermediate, and high risk, which correlate with outcome in radical prostatectomy and definitive radiation series (7, 8). More recently, clinical criteria have been introduced to predict pathologically “insignificant” prostate cancer, which is defined as clinical stage T1c, GS ≤6, disease in fewer than 3 biopsy cores, and less than 50% involvement in any core with PSA density <0.15 ng/mL/g (9–11). In addition, the Memorial Sloane-Kettering group identified high-risk features within the intermediate risk group, which correlated with decreased biochemical freedom from relapse, decreased local control, increased distant metastasis, and increased prostate-specific mortality. They created stratifications of favorable and unfavorable intermediate risk groups, assigning the unfavorable intermediate risk group to patients with primary Gleason pattern of 4, percentage of biopsy positive cores ≥50%, or multiple NCCN-defined intermediate risk factors (cT2b-c, PSA 10–20, or GS 7) (12).

The NCCN Prostate Cancer Panel recognized that heterogeneity exists within the previous risk stratification groups and has redesigned guidelines for prostate cancer by risk categories, which are linked to treatment paradigms with the hope that these updated risk groups can be used more accurately to begin discussing treatment options with prostate cancer patients. These updated NCCN guidelines, version 2.2014, stratify prostate cancer into five risk groups (very low, low, intermediate, high, and very high) utilizing GS, initial PSA, and T-stage (2, 13). These guidelines separate out “insignificant” very low risk prostate cancer and suggest raising the risk group depending on number of risk factors within each group. Clinical stage T2c is now considered intermediate risk by NCCN (2) compared to the D’Amico classification, which places T2c patients into the high risk category (7, 8).

We report an update of our previously published experience with CyberKnife SBRT (14), assessing efficacy and toxicity while exploring the new NCCN risk groups in a consecutively treated database of early stage prostate cancer. We have adopt the Memorial group’s favorable and unfavorable intermediate risk stratification but simplify the unfavorable intermediate group to patients who demonstrate multiple NCCN-defined intermediate risk factors to conform to the new NCCN guidelines.

## Materials and Methods

One hundred forty-two early stage prostate cancer patients received SBRT at the Philadelphia CyberKnife Center from 2007 through 2012. The median and mean follow-up were 38 and 39 months, respectively, with a range of 0–76 months. This IRB-approved retrospective study included updated information on 70 patients from our previous experience, in addition to 72 patients treated thereafter. SBRT was delivered using the CyberKnife (Accuray Inc., Sunnyvale, CA, USA) with MultiPlan inverse treatment planning and motion tracking of internal fiducials. Treatment planning began with transrectal or transperineal ultrasound-guided placement of four gold fiducials into the prostate. A CT scan (1.25-mm slice thickness) was obtained 10–14 days later to allow inflammation to subside and to ensure fiducials did not migrate. T2 weighted turbo spin-echo MRI was obtained for contouring the prostate and seminal vesicles after registration by fiducials with CT scan and T1 vibe fat suppression MRI.

**Figure 1:**
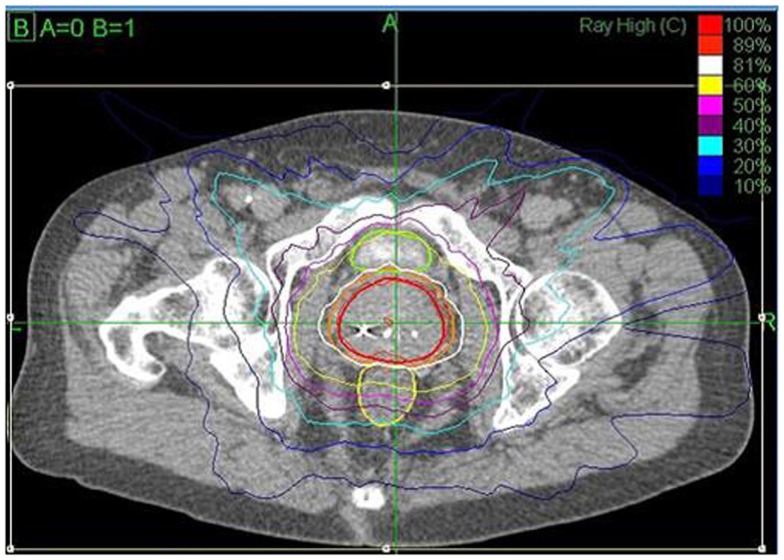
**CyberKnife SBRT treatment plan (36.25 Gy × 5 fractions prescribed to the 81% isodose line (white) with a 44.75 Gy maximum dose)**. Shown are the prostate (43.6 cm^3^, red) and PTV (77.8 cm^3^, orange). A bladder (green) volume of 1.66 cm^3^ received 37 Gy and 0.4 cm^3^ of the rectum (yellow) received 36 Gy.

The prostate, seminal vesicles, rectum, bladder, penile bulb, testes, and sigmoid colon were contoured (Figure 1). Urethra contouring was not required. The clinical target volume (CTV) was the prostate for low risk patients and the prostate plus 2-cm seminal vesicle base for intermediate/high risk patients. A total dose of 35, 36.25, or 37.5 Gy, delivered in five fractions, was prescribed to the planning target volume (PTV) that consisted of the CTV with a 5 mm margin in all directions except 3 mm posteriorly. Patients were typically treated over 8 days with a weekend off in between with an every other day treatment schedule. If necessary because of patient convenience or staff coverage, treatments were consecutive. Our first cohort of patients was treated with 35 Gy (*n* = 5) with subsequent dose escalation to 37.5 Gy (*n* = 30) per department protocol and review of radiobiology data available at the time. Most recent cohort was treated with a lower dose of 36.25 Gy (*n* = 107) after more published experience was available, as well as participation in a national protocol, which required that dose. The dose was not changed due to detected poor outcome or toxicity. The dosimetric goal was to cover at least 95% of the PTV with the prescribed dose normalized to the 75–85% isodose line (dose heterogeneity 17–33%). Less than 1 cm^3^ of rectum received 36 Gy, 50% of the prescribed dose could not cross the posterior rectal wall, and <10 cm^3^ of bladder received 37 Gy. Less than 50% of the penile bulb received 29.5 Gy and less than 1 cc of the sigmoid colon received 30 Gy. The average CTV and PTV were 56.9 (SD 26.1 cm^3^) and 98.3 cm^3^ (SD 38.6 cm^3^), respectively.

Orthogonal 120-kV X-ray image pairs were obtained throughout treatment for use in motion tracking. The real-time prostate position was locked-on by the relative fiducial position on the X-rays. For those patients with evenly distributed fiducials in the prostate quadrants, the prostate’s rotation was also tracked and corrections were made in real time.

PSA nadir was defined as the lowest PSA value following SBRT. Biochemical failure (BF) was assessed using the nadir + 2 (Phoenix) definition. Toxicity was assessed using the radiation therapy oncology group (RTOG) criteria; acute toxicity occurred within 3 months and late toxicity >3 months following treatment (15).

Pretreatment factors were assessed for each patient, which included standard risk factors of initial PSA, GS, and clinical stage. Patients were stratified into four risk groups as per NCCN 2.2014 since we had only two very high risk patients who were included with the high risk group. This stratification includes the NCCN “very low” risk group designation, which separates our earlier low risk group into two groups (very low and low). To explore group heterogeneity, we separated our previous intermediate risk group into favorable intermediate if there was one intermediate risk factor (Gleason 7 or clinical stage T2b/c or PSA > 10) or unfavorable intermediate if there was more than one intermediate risk factor. For the purpose of further analysis, the unfavorable intermediate group was then combined with the high risk group as per NCCN version 2.2014.

Kaplan–Meier estimates of freedom from biochemical failure (FFBF) were used to describe the patients overall, and comparisons were accomplished using log-rank statistics (16, 17). Simple Cox proportional hazards regression modeling was used to estimate hazard ratios for categorical variables of interest. The overall sample was described using measures of central tendency (mean and median) and variation (standard deviation, IQR). Descriptive statistics were used to describe toxicity within each of the treatment groups.

## Results

### Patient and treatment characteristics

One hundred and forty-two consecutive patients were analyzed with pretreatment and treatment factors described in Table 1. NCCN risk groups are depicted in Table 1 with either three (D’Amico classification) or five strata (NCCN version 2.2014 with intermediate risk group stratification and no high risk stratification). Androgen deprivation therapy (ADT) in the form of luteinizing hormone-releasing hormone agonist was used in 28.2% of patients (11.5% of the low risk, 37.7% of the intermediate risk and 50% of the high risk). The use of ADT was dependent on individual urologist and radiation oncologist preference. Selected very low and low risk patients received ADT to shrink the prostate before SBRT.

**Table 1:** **Patient and tumor characteristics**.

**Age at diagnosis**	**Years**	
Median (IQR)	67 (61,73)	
Range	44–88	
**Pretreatment PSA**	**ng/mL**	
Mean (SD)	8.1 (7.7)	
Median (IQR)	5.7 (4.4,8.3)	
**Pretreatment PSA**	**Number of patients**	**Percent**
<10 ng/mL	115	81
10–20 ng/mL	19	13
>20 ng/mL	8	6
**T-stage**	**Number of patients**	**Percent**
T1c	106	75
T2a	19	13
>T2a	17	12
**Gleason score**	**Number of patients**	**Percent**
5–6	76	53
7	54	38
8+	12	9
**Risk Group (3)**	**Number of patients**	**Percent**
Low	61	43
Intermediate	63	44
High	18	13
**Risk group (5)**	**Number of patients**	**Percent**
Very low	28	20
Low	33	23
Favorable intermediate	50	35
Unfavorable intermediate	13	9
High	18	13
**Dose group**	**Number of patients**	**Percent**
Low	112	79
High	30	21
**Hormone treatment**	**Number of patients**	**Percent**
No ADT	102	72
ADT	40	28

Patients who received 35 or 36.25 Gy were termed the “low-dose group” and those who received 37.5 Gy the “high-dose group.” Most patients (57%) received their treatment over 5–8 days. The remainder completed treatment between 9 and 19 days with one non-compliant patient receiving his final fraction several weeks later, all due to poor adherence to their schedule. Treatment was delivered with an average of 187 (SD = 26) non-coplanar beams. X-ray images were taken every three to five beams to track the prostate’s movement.

### PSA response and biochemical control

**Figure 2:**
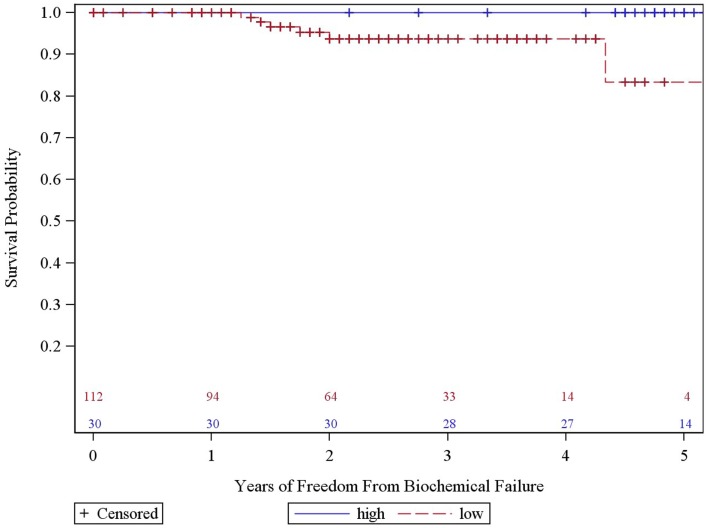
**Years of freedom from biochemical failure by dose for all patients**.

The overall 5-year actuarial FFBF for the whole group was 92.7% (Table 2). Six patients out of the entire cohort of 142 patients, all in the low dose group, experienced BF; two were high risk, three intermediate risk, and one low risk. The low-dose and high-dose groups’ median PSA nadirs were 0.3 and 0.1 ng/mL, respectively. Five year actuarial FFBF was 100% for the high dose group and 93.75% for the low dose group, *p* = 0.05, hazard ratio of 11.0 for low compared to high dose (Table 2, Figure 2).

**Table 2:** **Freedom from biochemical failure stratified by risk and descriptive statistics**.

		1 year (%)	2 year (%)	3 year (%)	4 year (%)	5 year (%)	*P*-value	Hazard ratio
All patients		100.0	95.5	95.5	95.5	92.7		
	High risk	100.0	83.9	83.9	83.9	83.9	0.34	9.3
	Intermediate unfavorable	100.0	90.0	90.0	90.0	90.0		6.8
	Intermediate favorable	100.0	95.2	95.2	95.2	95.2		3.1
	Low risk	100.0	100.0	100.0	100.0	91.7		2.4
	Very low risk	100.0	100.0	100.0	100.0	100.0		1.0
	High risk	100.0	83.9	83.9	83.9	83.9	0.14	7.0
	Intermediate risk	100.0	94.2	94.2	94.2	94.2		2.5
	Low risk	100.0	100.0	100.0	100.0	94.4		1.0
Combined risk groups	High	100.0	86.7	86.7	86.7	86.7	0.07	4.0
	Very low/low/intermediate	100.0	97.9	97.9	97.9	94.4		1.0
Pretreatment PSA	<10	100.0	95.58	95.58	95.58	92.04	0.86	1.0
	10–20	100.0	92.9	92.9	92.9	92.9		1.7
	>20	100.0	100.0	100.0	100.0	100.0		1.8
T-stage	T1c	100.0	97.8	97.8	97.8	94.2	0.19	1.0
	T2a	100.0	85.7	85.7	85.7	85.7		4.7
	Other (T2b +)	100.0	90.0	90.0	90.0	90.0		3.5
Gleason Score	5–6	100.0	100.0	100.0	100.0	95.5	**0.03**	1.0
	7	100.0	93.4	93.4	93.4	93.4		3.5
	8+	100.0	76.2	76.2	76.2	76.2		12.5
Dose group	Low	100.0	93.8	93.8	93.8	93.8	**0.05**	11.0
	High	100.0	100.0	100.0	100.0	100.0		1.0
ADT	No ADT	100.0	95.0	95.0	95.0	90.8	0.48	1.6
	ADT	100.0	96.7	96.7	96.7	96.7		1.0

*Bold font indicates significant *p*-value*.

**Figure 3:**
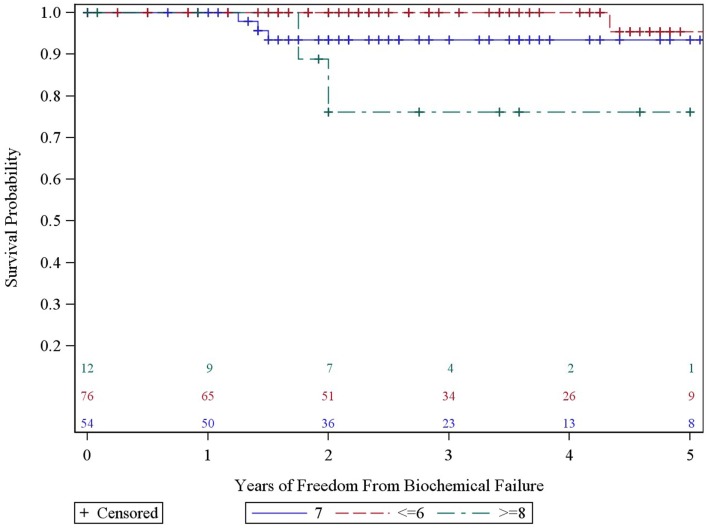
**Years of freedom from biochemical failure by Gleason score for all patients**.

We analyzed the heterogeneity of intermediate risk prostate cancer by stratifying into an intermediate favorable and unfavorable as described in the methods with a 5.2% decrement in 5-year actuarial FFBF from 95.2 to 90.0%. We then compared the intermediate unfavorable with high risk and found a 6.1% decrement in 5-year actuarial FFBF from 90 to 83.9%. We then grouped the intermediate unfavorable with the high risk group as per version 2.2014 NCCN guidelines with 5-year actuarial FFBF of 100% for very low risk, 91.7% for low risk, 95.2% for intermediate risk, and 86.7% for high risk (Table 2). Previous NCCN risk categories of low, intermediate, and high 5-year actuarial FFBF are presented also in order to compare to other contemporary series of prostate cancer SBRT (Table 2). Since there was little difference in outcome in our data base between very low, low, and intermediate NCCN version 2.2014 risk categories, they were grouped and compared to high risk with 5 year actuarial FFBF of 94.4% for very low/low/intermediate and 86.7% for high (*p* = 0.07, Table 2). A statistically significant decrement in 5-year actuarial FFBF was noted with increasing GS from 95.45 to 93.4% for GS 5/6 and 7 compared with 76.2% for GS ≥ 8, *p* = 0.03, hazard ratio was 12.5 for GS ≥ 8 vs. 3.5 for GS 7 vs. 1.0 for GS ≤ 6 (Table 2, Figure 3). Pretreatment PSA, T-stage, age, use of ADT, and risk stratification groups were not significant with respect to 5-year FFBF.

Multivariate analysis evaluated the most significant factors in univariate analysis including risk stratification groups, GS, and dose. GS and dose were the most predictive factors in multivariate analysis but did not reach statistical significance at the *p* < 0.05 level. Of note, with only six failures, we only have the power to detect a single significant predictor of failure in the multivariate analysis.

### Toxicity

Acute grade II GU toxicities were seen in 28% (*n* = 32/113) and grade III GU toxicities were seen in 2% (*n* = 3/142). Two of the three grade III acute GU toxicities manifested as frequency at least every hour and the other experienced gross hematuria. The patient who experienced gross hematuria had grade II baseline score with multiple urinary tract infections and transurethral resection of the prostate (TURP) prior to CyberKnife. All three patients’ toxicities resolved within 3 months of treatment. However, one case of frequency at least every hour reappeared as a late toxicity 6 months after treatment. Acute gastrointestinal (GI) toxicity was limited to 4% (5/141) grade II toxicity with no acute grade III GI toxicity.

At most recent follow-up, persistent toxicities were limited to grade II GU of 14% (18/132) and grade II GI of 3% (4/141). For these persistent GI toxicities, there was no difference between dose groups; however, for persistent grade II GU toxicities, the low dose group observed less than the high dose group at 10 (10/102) and 27% (8/30), respectively. Of note there were three late grade III GU toxicities 2% (*n* = 3/142), of which all three grade III patients were in the high dose group. One patient with a 162 cm^3^ CTV (the largest prostate in our patient sample) had acute grade III frequency that resolved within 2 weeks of alpha-blocker and prophylactic antibiotic treatment, but recurred 6 months later. His symptoms improved to grade II immediately after TURP, which was preformed 13 months after SBRT. Pathology of the resected tissue was negative for tumor. The second patient with late grade III GU toxicity experienced urinary retention. He had benign prostate hypertrophy (BPH) and grade II symptoms at baseline. At 14 months following SBRT, his symptoms progressed to grade III then completely subsided following laser TURP. All three grade III toxicities resolved at last follow to grade II or below.

Erectile dysfunction (ED) was assessed for the 48 patients who were potent before SBRT and did not receive ADT. At last follow-up, 13% lost the ability to achieve erections strong enough for penetration and required ED medication for intercourse. Six percent of patients who were potent before SBRT and did not receive ADT developed ED refractory to medical treatment.

## Discussion

In this updated study, we double the original number of patients and again report excellent FFBF rates with acceptable toxicity. The observed FFBF rates are concordant with other published SBRT outcomes for localized prostate cancer (Table 3) (3–5, 18).

**Table 3:** **SBRT publications for treatment of prostate cancer with 5 year actuarial %FFBF**.

Study author	Patient population	Number of patients	Dose	Median follow-up (months)	5-year %FFBF		
					Low	Intermediate	High
Freeman and King (5)	Low risk only, 2 institutions	41	35Gy/5, 36.25Gy/5	60	93	–	–
Kang et al. (18)	3 risk groups, single institution	44	32Gy/4, 34 Gy/4, 36Gy/4	40	100	100	91
King et al. (6)	3 risk groups, 8 institutions	1100	36.25Gy/4–5	36	95	84	81
Katz et al. (19)	3 risk groups, single institution	304	35Gy/5, 36.25Gy/5	60	97	91	74
Current study	3 risk groups, single institution	142	35Gy/5, 36.25Gy/5, 37.5Gy/5	38	94	94*	84

*Seven patients with T2c only included in intermediate group as per new NCCN guidelines.

To our knowledge, this is the first SBRT prostate cancer data base reporting outcome stratified retrospectively by the new version 2.2014 NCCN risk groups. In addition, we compare the prognostic value of other known factors in conjunction with NCCN risk groups in univariate and multivariate analysis. The current NCCN risk strategy separates out a very low risk group using many pathologic features in addition to the classic factors of low risk disease and separates out an unfavorable intermediate risk group to be combined with the high risk group. Our data suggest very little decrement in 5-year FFBF for very low, low, and favorable intermediate groups until at least two intermediate risk factors are present (unfavorable intermediate), which is grouped with high risk in the version 2.2014 NCCN guidelines.

Our data also suggest that GS may be the most important prognosticator being even more important than risk group stratification. The hazard for GS 8 is over 12 fold than that of GS 5–6 and 9 fold than that of GS 7 in our series with *p* = 0.03. Katz et al. observed a decrease in 5 year FFBF for patients within the intermediate risk group with GS 4 + 3 vs. all others from 95 to 84% (4). Katz et al. more recently reported their unfavorable intermediate and high risk groups utilizing the NCCN version 2.2014 criteria. Comparable to our series, only 47% of their high risk patients received androgen deprivation with no difference in FFBF noted in multivariate analysis. PSA was significant for outcome while GS was not which differs from our series (19).

In our previous experience with 3-year actuarial FFBF rates, we noted a statistically significant dose response for the intermediate and high risk patients (14). In the current study, which includes a larger sample size and longer follow-up, we continue to observe a significant increase in 5-year FFBR between high and low dose groups (*p* = 0.05). The sample size of the high dose group (*n* = 30) is about one-fourth of the size of the low dose group; nevertheless, no BF occurred in the high-dose group despite the longer median follow-up (57 vs. 33 months). This may come at the cost of increased persistent late GU toxicity with 27% grade II toxicities (compared to 10% in the low dose group). However, it is also important to note that no grade III GU toxicities persist at most recent follow-up.

A recently published multi-institutional consortium from eight independent phase 2 studies has pooled together 1100 patients with localized prostate cancer (3, 6). Patients had been treated definitively with CyberKnife SBRT, having a median follow-up of 36 months and median dose of 36.25 Gy in 4–5 fractions. The risk stratification consisted of 58% low risk, 30% intermediate risk, and 11% high risk. They reported a 5-year FFBF 95, 84, and 81%, respectively. Their overall 5-year FFBF rate was 93%, with 49 total patients (4.9%) experienced failure. This experience parallels our own, despite having a larger percentage of high and intermediate risk patients in the current series. There was no observed dose response among the range of doses used in the consortium of 35–40 Gy. In addition to this analysis, they used a validated nomogram to compare outcomes of SBRT to radical prostatectomy, external beam radiotherapy, and permanent brachytherapy at 5 years. Although our current study is retrospective, it is encouraging to note these three conventional modalities have historically shown FFBF rates comparable to CyberKnife SBRT in our study as well as the aforementioned consortium of eight phase 2 studies.

Our late grade III urinary toxicity is similar to that reported in the literature with no grade III GI toxicity (4, 5, 18–21). In our experience, dose may increase this risk since all three patients in our series with late grade III urinary toxicity were in the high dose group of 37.5 Gy. This has been also reported by Katz et al. where all patients in his series with grade III toxicity were in the 36.25 Gy group compared with his 35 Gy group (4).

Radiation therapy oncology group 0938 is a randomized phase II trial assessing hypofractionated regimens of 36.25 Gy in five fractions of 7.25 Gy, and 51.6 Gy in 12 fractions of 4.3 Gy (22). The patients in this protocol are favorable risk, and hypofractionated schemes were delivered via linear accelerator, CyberKnife, or proton therapy. The RTOG protocol included a fractionation scheme (36.25 Gy in five fractions at 7.25 Gy), which was prescribed for 75% of patients in our study. Although the patient population in this protocol are all “favorable risk,” the primary endpoint is first-year health-related quality of life (HRQOL) measured by Bowel and Urinary domains of the Expanded Prostate Cancer Index Composite (EPIC) instrument; therefore, the results will be meaningful from a toxicity standpoint and applicable to less favorable risk patients (i.e., intermediate and high risk patients). Favorable HRQOL and EPIC scores may further validate the dose of 36.25 Gy in five fractions as the new standard. Fortunately, this protocol met accrual goals and closed February 2014.

In conclusion, SBRT could be considered an appropriate alternative to other standard radiation treatment modalities for early stage prostate cancer with excellent 5-year FFBF rates and low toxicity. Clinical expertise is required, but SBRT can be safely delivered in a community practice setting. The NCCN version 2.2014 further refines the risk strata, which may help guide patients treatment choices. GS may be the most important and simple prognosticator for risk assessment. Further study is warranted for unfavorable intermediate and high risk patients whose numbers are limited in most SBRT series. The role of dose escalation is unclear due to conflicting reports on the benefit on FFBF and risk of toxicity and also warrants additional study.

## Conflict of Interest Statement

The authors Dr. Rachelle Lanciano, Dr. John Lamond, Dr. Stephen Arrigo, and Dr. Luther W. Brady each own a small percentage of the single unit CyberKnife at the Philadelphia CyberKnife Center. The other co-authors declare that the research was conducted in the absence of any commercial or financial relationships that could be construed as a potential conflict of interest.
